# Ilio-psoas hematoma in the intensive care unit: a multicentric study

**DOI:** 10.1186/s13613-016-0106-z

**Published:** 2016-01-19

**Authors:** J. F. Llitjos, F. Daviaud, D. Grimaldi, S. Legriel, J. L. Georges, E. Guerot, J. P. Bedos, J. Y. Fagon, J. Charpentier, J. P. Mira

**Affiliations:** Medical Intensive Care Unit, Cochin Hospital, Groupe Hospitalier Cochin Broca Hôtel-Dieu, Assistance Publique des Hôpitaux de Paris, 27 rue du Faubourg Saint-Jacques, 75014 Paris, France; Faculté de Médecine, Université Paris Descartes, Sorbonne Paris Cité, 15 rue de l’Ecole de Médecine, 75006 Paris, France; Intensive Care Unit, Hôpital de Versailles - Site André Mignot, 177 rue de Versailles, 78150 Le Chesnay Cedex, France; Cardiology, Hôpital de Versailles - Site André Mignot, 177 rue de Versailles, 78150 Le Chesnay Cedex, France; Medical Intensive Care Unit, Hôpital Européen Georges Pompidou, AP-HP, Paris, France

**Keywords:** Ilio-psoas hematoma, Psoas, Intensive care, Anticoagulant therapy, Reversal

## Abstract

**Background:**

Clinical features and outcomes of patients with spontaneous ilio-psoas hematoma (IPH) in intensive care units (ICUs) are poorly documented. The objectives of this study were to determine epidemiological, clinical, biological and management characteristics of ICU patients with IPH.

**Methods:**

We conducted a retrospective multicentric study in three French ICUs from January 2006 to December 2014. We included IPH diagnosed both at admission and during ICU stay. Surgery and embolization were available 24 h a day for each center, and therapeutic decisions were undertaken after pluridisciplinary discussion. All IPHs were diagnosed using CT scan.

**Results:**

During this period, we identified 3.01 cases/1000 admissions. The mortality rate of the 77 included patients was 30 %. In multivariate analysis, we observed that mortality was independently associated with SAPS II (OR 1.1, 95 % CI [1.013–1.195], *p* = 0.02) and with the presence of hemorrhagic shock (OR 67.1, 95 % CI [2.6–1691], *p* = 0.01). We found IPH was related to anticoagulation therapy in 56 cases (72 %), with guideline-concordant reversal performed in 33 % of patients. We did not found any association between anticoagulant therapy type and outcome.

**Conclusion:**

We found IPH is an infrequent disease, with a high mortality rate of 30 %, mostly related to anticoagulation therapy and usually affecting the elderly. Management of anticoagulation-related IPH includes a high rate of no reversal of 38 %.

## Background

Ilio-psoas hematoma (IPH) is defined as a spontaneous or traumatic retroperitoneal collection of blood involving the ilio-psoas muscle. Even if the precise occurrence of spontaneous IPH remains uncertain, mostly because a few studies are available, incidence of retroperitoneal bleeding in patients undergoing anticoagulation has been reported ranging from 0.1 to 0.6 % [1].

Risk factors for spontaneous retroperitoneal hematoma are anticoagulation therapy, the elderly and hemodialysis [2]. Whereas precise pathogenesis and pathophysiology of retroperitoneal bleeding is unknown, it is hypothesized that retroperitoneal microvascular atherosclerosis could increase sensitivity to rupture, with an involvement of large vessels if stretched by the enlarging hematoma. Anticoagulation and microtrauma such as cough or vomiting could also lead to retroperitoneal bleeding. Nevertheless, theses theories are not confirmed on histology.

Whether incidental diagnosis is frequent, clinical presentation depends on the severity of the hemorrhage, varying from compressive symptoms, such as nerve palsy or ischemia, to lower pain and/or anemia [3]. CT scan is the main tool for diagnosis, providing useful information on volume, spatial extent, compressive complications or active bleeding [4, 5].

Incidence of IPH in the intensive care is unknown. Most of the time, these patients usually need hemodynamic and/or respiratory support and blood transfusion [6]. Given the lack of evidences, there is no consensus on therapeutics, and each decision, i.e., conservative treatment, embolization, surgical or CT scan-guided hematoma’s drainage, is made by weighting risks and benefits [7].

We conducted a multicentric retrospective study of patients with a diagnosis of spontaneous IPH in the intensive care in order to assess incidence, fate, management and outcome of these patients.

## Methods

### Patients

We retrospectively included patients with IPH admitted in three French academic intensive care units (Cochin Hospital, Versailles Hospital and Georges Pompidou European Hospital) from January 2006 to December 2014. There is no reference center for IPH within the Paris metropolitan area, and these three centers were selected because they manage both medical and surgical patients and because surgery and embolization were available 24 h a day for each center. There was no consensual guideline to manage IPH among the three hospitals. Patients were evaluated, and the severity was assessed by a senior intensivist in the emergency department or in a medical department of the hospital for each center prior to ICU admission.

### Data collection

For every center, we screened all admitted patients from January 2006 to December 2014 and cases were identified using informatics medical charts with the following keywords “psoas,” “iliac,” “ilio-psoas,” “bleed” and “hematoma,” We included every patient of 18 years old or more presenting a non-traumatic IPH, and we excluded traumatic causes of IPH. Patients with IPH admitted from other centers in order to perform open surgery and/or embolization were excluded. Data retained were patient’s demographics, initial prognosis, severity at admission, medical history, laboratories and imaging investigations, and intra-hospital course. Patient’s characteristics and preexisting conditions were abstracted from the medical charts. Simplified Acute Physiology Score II (SAPS II), Sequential Organ Failure Assessment (SOFA) score, heart rate, mean arterial pressure and temperature were computed at diagnosis of the IPH. Hemorrhagic shock was defined according to international criteria as hemodynamic instability defined by systolic arterial blood pressure (SAP) < 90 mmHg or signs of shock and the presence of a life-threatening bleeding or bleeding that compromises vital function [8]. Liver failure was defined as a factor *V* < 50 % or the presence of jaundice and acute kidney injury as an increase in serum creatinine by 26.5 μmol/l within 48 h or an increase up to 1.5 times baseline [9]. IPH was related to anticoagulant treatment in patients treated with vitamin K antagonist (VKA), low molecular weight heparin (LMWH) or unfractionned heparin (UH) regardless of the presence of an over-anticoagulation, as defined in guidelines [10]. CT scan with contrast enhancement was performed for each patient. Blood and derived products were transfused according to physician judgment. Therapeutic decisions were undertaken after pluridisciplinary discussion involving surgeons and intensivists. Surgery and embolization were available 24 h a day for each center.

### Statistical analysis

Quantitative parameters and continuous data are presented as median and interquartile range (IQR, 25th–75th percentile) and were compared between groups using nonparametric *U* Mann–Whitney test. Qualitative parameters are presented as counts and percentage. Categorical variables were compared using the Chi-square test or Fischer’s exact test, as appropriate. A multivariate analysis was performed to identify independent factors using a binomial logistic regression. Variables included in the multivariate analysis were those significant (*p* < 0.05) in univariate analysis, and we calculated the odds ratio (OR) associated and its 95 % confidence interval (CI). All P value was two-tailored, and statistical significance was set at *p* < 0.05. All statistical analyses were carried out with SPSS^®^ version 20.0.

## Results

**Fig. 1:**
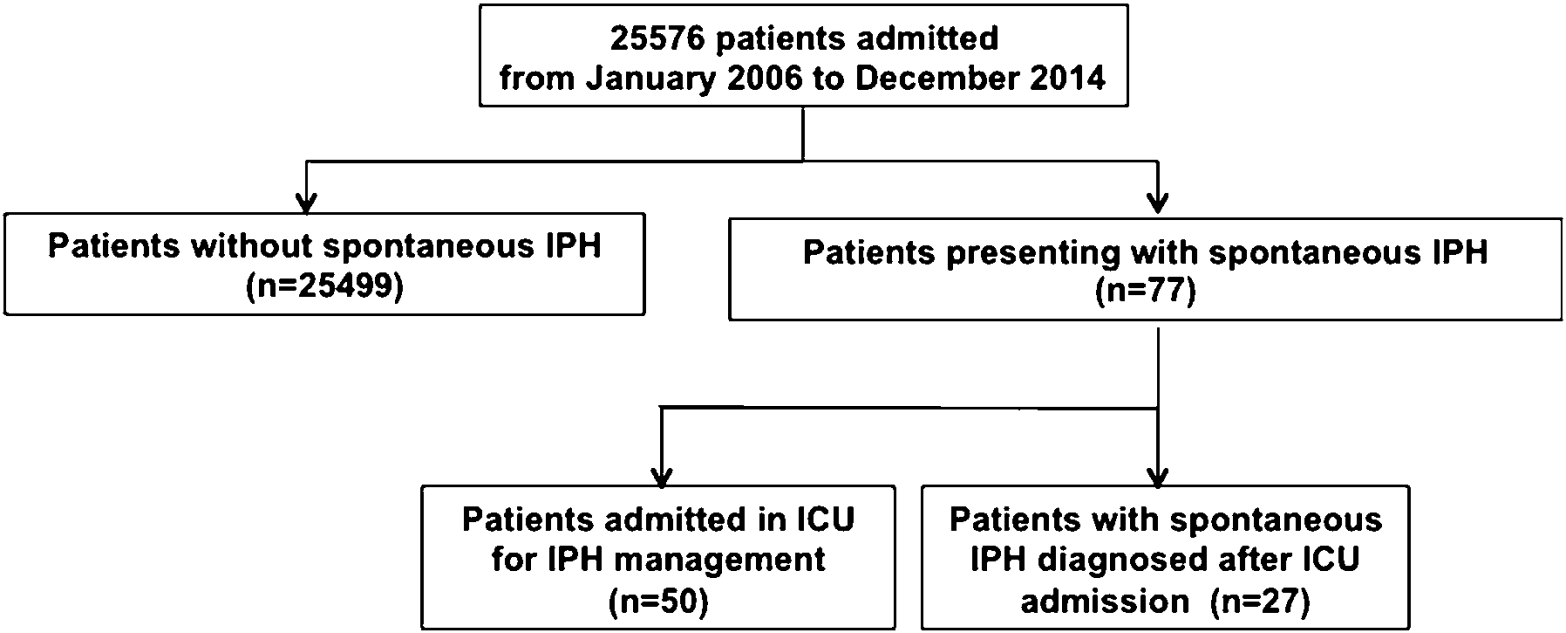
Flowchart of the study: patients with ilio-spoas hematoma

The study flowchart is shown in Fig. 1. Between January 2006 and December 2014, among 25,576 patients admitted in ICU, we retained 77 patients having an ilio-psoas hematoma, corresponding to 3.01 cases/1000 admissions during this period. Main clinical patient’s characteristics and preexisting conditions at ICU admission are summarized in Table 1. Fifty patients were admitted in ICU for IPH management, whereas the diagnosis of IPH was made in 27 ICU patients previously hospitalized, with a median time of 7 days (IQR 3.5–17). Median age at diagnosis was 72 years (IQR 64.8–79), and 31 patients were female (40 %). Thirty-four patients presented with hemorrhagic shock (44 %) at diagnosis. CT scan was performed in every patient, with an active arterial bleeding diagnosed in 29 % of patients (*n* = 22) and compression of adjacent structures diagnosed in 34 % of patients (*n* = 26). The mortality rate was 30 % (*n* = 23), with no difference across centers.

**Table 1:** Clinical characteristics at diagnosis

Clinical characteristic at diagnosis	All (*n* = 77)	Died (*n* = 23)	Alive (*n* = 54)	*p* value	OR	95 % CI of OR
Age, years	72 (64.8–79)	71 (69–81)	72 (63–78)	ns		
Female sex, *n* (%)	31 (40)	10 (43)	21 (39)	ns		
Preexisting conditions						
Hypertension, *n* (%)	45 (58)	17 (74)	28 (52)	ns		
Smoking, *n* (%)	27 (35)	5 (22)	22 (41)	ns		
Body mass index, kg/m^2^	27 ± 6	27 ± 4	27 ± 6	ns		
Chronic cardiac failure, *n* (%)	24 (31)	6 (26)	18 (33)	ns		
Diabetes, *n* (%)	10 (13)	4 (17)	6 (11)	ns		
Renal insufficiency, *n* (%)	18 (23)	4 (17)	14 (26)	ns		
Peripheral vascular disease, *n* (%)	14 (18)	6 (26)	8 (15)	ns		
Cirrhosis, *n* (%)	2 (2)	1 (4)	1 (2)	ns		
Cancer or hematological malignancy, *n* (%)	25 (32)	7 (30)	18 (33)	ns		
Remission, *n*	9	3 (13)	6 (11)	ns		
Evolutive, *n*	16	4 (17)	12 (22)	ns		
Glasgow Coma Scale	15 (11.5–15)	11 (3–15)	15 (14–15)	0.04		
SOFA score	6 (3.5–10)	10 (5–15)	5 (3–8)	<0.001		
SAPS II score	56 (36.5–59)	75 (57–95)	48 (31–60)	<0.001		
Clinical presentation, *n* (%)						
Hemorrhagic shock	34 (44)	22 (95)	12 (22)	<0.001	77	(9.3–631)
Compressive symptoms	20 (25)	5 (21)	15 (27)	ns		
Abdominal bruises	23 (29)	6 (26)	17 (31)	ns		
Incidental diagnosis on CT scan	11 (14)	3 (13)	8 (15)	ns		
Heart rate, beats per minute	94 ± 25	100 ± 32	93 ± 22	ns		
Mean arterial pressure, mmHg	83 ± 20	71 ± 19	83 ± 20	0.02		
Temperature, °C	37 (36–37.4)	36.1 (35–37.2)	37 (36.7–37.6)	0.01		
Hemoglobin. g/dL	7.5 (6.4–9.2)	7.3 (5.8–8.7)	7.7 (6.6–9.4)	ns		
Lactate at admission, mmol/L	2.2 (1.4–7.3)	7.8 (2.6–11.8)	1.9 (1.1–3.8)	<0.001		

*OR* odds ratio, *95* *% CI* 95 % confidence interval, *SOFA* Sequential Organ Failure Assessment score, *SAPS II score* Simplified Acute Physiology Score II

Data concerning management of patient are summarized in Table 2. Vasopressive support and mechanical ventilation were more frequently performed in died patients than in survivors (87 vs. 39 %, *p* > 0.001 and 96 vs. 50 %, *p* > 0.001, respectively). Whether conservative treatment was less frequently performed in died patients when compared to the survivors (78 vs. 48 % respectively, OR 0.24, 95 % CI [0.08–0.68], they underwent more frequently surgery (26 vs. 2 % respectively, OR 18.7, 95 % CI [2.1–166.5]). In multivariate analysis (Table 3), we observed that mortality was independently associated with SAPS II (OR 1.1, 95 % CI [1.013–1.195], *p* = 0.02) and with the presence of hemorrhagic shock (OR 67.1, 95 % CI [2.6–1691], *p* = 0.01).

**Table 2:** Management in the ICU of the overall cohort of IPH

Management in the ICU	All (*n* = 77)	Died (*n* = 23)	Alive (*n* = 54)	*p* value	RR	95 % CI of OR
Acute heart failure, *n* (%)	11 (14)	6 (26)	5 (9)	ns		
Vasopressive support during the first 24 h, *n* (%)	39 (51)	21 (91)	18 (33)	<0.001	17.8	(3.7–84.2)
Norepinephrine, *n*	25	16	9			
Epinephrine, *n*	11	5	6			
Dobutamine, *n*	5	2	3			
Liver failure (factor *V* < 50 % or jaundice), *n* (%)	16 (21)	12 (52)	4 (7)	<0.001	1.6	(3.6–50)
Acute kidney injury, *n* (%)	42 (55)	19 (83)	23 (43)	0.001	6.4	(1.9–21.3)
Dialysis, *n*	19	10	9			
Hemofiltration, *n*	4	2	2			
Renal recovery at ICU discharge, *n*	14	1	13			
Duration of renal replacement therapy, days	15.8 ± 16.9	18.1 ± 21.1	5.3 ± 5.4	0.01		
Mechanical ventilation, *n* (%)	49 (63)	22 (96)	27 (50)	<0.001	22	(2.7–175)
Duration of mechanical ventilation, days	3 (0–10.5)	6 (2–21)	1 (1–8)	0.002		
Packed red blood cell transfusion, units	4 (2–6)	6 (3–8)	3(2–5)	0.002		
Conservative treatment, *n* (%)	53 (69)	11 (48)	42 (78)	0.007	0.24	(0.08–0.68)
Surgery, *n* (%)	7 (9)	6 (26)	1 (2)	0.002	18.7	(2.1–166.5)
Embolization, *n* (%)	17 (22)	6 (26)	11 (20)	ns		

*OR* odds ratio, *95* *% CI* 95 % confidence interval

**Table 3:** Multivariate predictors of intensive care unit mortality in patients with IPH

Variables	*b*	se	Wald test	*df*	OR	95 % CI for OR	*p* value
Hemorrhagic shock	4.206	1.646	6.527	1	67.1	2.663–1691	0.011
SAPS II score	0.095	0.042	5.092	1	1.1	1.013–1.195	0.024
Lactate	0.262	0.149	3.091	1	1.3	0.97–1.741	0.079
Duration of mechanical ventilation	0.052	0.032	2.615	1	1.054	0.989–1.123	0.106
Number of packed red blood cells transfused	0.531	0.287	3.413	1	1.701	0.968–2.988	0.065
Conservative treatment	0.207	1.101	0.035	1	1.23	0.142–10.65	0.851

*b* slope, *se* standard error, *df* degrees of freedom, *OR* odds ratio, *95* *% CI* 95 % confidence interval

We found IPH was related to anticoagulant therapy in 56 cases (72 %), with 21 cases of vitamin K antagonists (VKA) therapy, 23 cases of unfractionated heparin (UH) therapy and 12 cases of low molecular weight heparin (LMWH) therapy. Patients were on anticoagulant therapy for the following reasons: atrial fibrillation (*n* = 18), deep venous thrombosis (*n* = 14), pulmonary embolism (*n* = 13), prosthetic heart valve (*n* = 7), stroke (*n* = 1) and acute myocardial infarction (*n* = 3). Among anticoagulated patients, 11 patients were previously treated with aspirin and 8 patients received a combination of aspirin and clopidogrel for coronary artery disease, with no difference in mortality. We found no difference on mortality in patients having or not an anticoagulation treatment.

Regardless of administration time, the guideline concordant recommended co-administration of vitamin K and prothrombin complex concentrate at appropriate doses in VKA-related hematoma accounted for 33 % of patients in the whole cohort (Table 4). Protamine reversal was performed in 13 % (*n* = 3) of patients with UH-related hematoma and in 1 survivor patient with LMWH-related hematoma. Among 34 patients with hemorrhagic shock at admission, 24 are related to anticoagulation therapy and only 9 (37 %) are reversed.

**Table 4:** Anticoagulated-related hematoma: reversal management

Anticoagulated-related hematoma	All	Died	Alive	*p* value
VKA-related hematoma, *n*	21	5	16	
Reversal management				
No reversal, *n* (%)	8 (38)	1 (20)	7 (44)	ns
No guideline-concordant reversal, *n* (%)	6 (29)	2 (40)	4 (25)	ns
Guideline-concordant reversal, *n* (%)	7 (33)	2 (40)	5 (31)	ns
INR at diagnosis				
Normal (INR < 1.5), *n* (%)	0 (0)	0 (0)	0 (0)	ns
Therapeutic (INR between 1.5 and 4), *n* (%)	8 (38)	3 (50)	5 (33)	ns
Supratherapeutic (INR > 4), *n* (%)	9 (57)	1 (17)	8 (53)	ns
Missing values, *n* (%)	4 (19)	2 (33)	2 (13)	ns
Unfractionned heparin-related hematoma, *n*	23	6	17	
Protamine reversal, *n* (%)	3 (13)	1 (17)	2 (12)	ns
Low molecular weight heparin-related hematoma, *n*	12	3	9	
Protamine reversal, *n* (%)	1 (8)	0 (0)	1 (11)	ns

## Discussion

In the following cohort, we found that IPH is an infrequent pathology with 77 cases identified on 25,576 admissions between January 2006 and December 2014. To our knowledge, this is the first report of incidence of admission in ICU. In a large observational cohort study of severe hemorrhage in 822 VKA-treated patients, bleeding was located in deep muscle and in the subperitoneal area in 13 % [11]. Moreover, in a retrospective study of 180 patients presenting a spontaneous retroperitoneal hematoma, 62 % of patients were admitted in ICU [12]. The relative low proportion of IPH among global ICU patients we observed in our study may be partly explained by the fact that retroperitoneal bleeding accounts for a small part of overall severe bleeding, and patients are scarcely admitted in ICU.

We found IPH was related to VKA therapy in 21 patients (27 %), with no reversal treatment in 38 % and with no guideline-concordant reversal in 29 %. According to guidelines, reversal treatment of severe bleeding under VKA therapy consists in restoring iso-coagulation with an INR < 1.5 as soon as possible. It is recommended to perform a prompt reversal using at least 20 IU/kg factor IX equivalent prothrombin complex concentrate (PCC) and at least 5 mg of vitamin K performed within a predefined time frame of 8 h after admission [10]. Regardless of administration time, in our study the guideline-concordant co-administration of PCC and vitamin K was performed in one-third of patients. It is well established that many factors may influence the implementation of a guideline in practice such as individual patient, the individual healthcare provider and the organizational context [13]. In the context of our study, various barriers may explain this low adherence rate to guideline-concordant reversal such as the fear of thrombosis in case of normal INR or the unawareness of guidelines by the physicians. Among 34 patients with hemorrhagic shock at admission, 24 are related to anticoagulation therapy and only 9 (37 %) are reversed, thus suggesting that reversal does not depend on the practitioner’s perception of the severity. This low proportion of reversal in anticoagulated patients presenting with hemorrhagic shock may explain in part their frequent fatal outcome. In multivariate analysis, the presence of hemorrhagic shock is independently associated with mortality. This association highlights the fact that prognosis is intrinsically and closely linked to the bleeding and emphasizes the importance of a rapid and appropriate management of the blood loss.

This study shows that IPH is associated with a 30 % mortality rate in the ICU. Whether a precise description of usual mortality of IPH in ICU is not reported in the literature, this can be roughly approximated by some studies. Mortality ranges from 12 to 19 % [12, 14], with various admission rates in ICU (62 and 40 %, respectively). In 29 patients, Guivarc’h et al. reports a mortality of 3 %, with surgery performed in 88 % of cases [15]. However, in this cohort, only 28 % were under excessive anticoagulation and only 4 patients were hemodynamically unstable. Beside the ICU, mortality of deep-muscle VKA-related hematoma in the emergency department can be as low as 4 % [11]. We herein observe a higher mortality, thus suggesting patients admitted in ICU have a worse prognosis at admission than patients previously reported in the literature. Consistently, classical markers of severity, such as lactate level, SAPS II or SOFA score, are high in non-survivor patients in our cohort.

Management of life-threatening psoas hematoma, and more widely of soft tissue hemorrhage, in anticoagulated patients remains controversial. Whether conservative treatment is recommended in hemodynamically stable patient with no evidence of ongoing bleeding, the usefulness of embolization or surgery in case of bleeding is far from being demonstrated. Surgery seems to be indicated when the patient remains unstable despite medical resuscitation, if interventional radiology is not successful or unavailable or in case of abdominal compartment syndrome [2]. Open surgery consists in identification and control of the bleeding source, evacuation of the hematoma and packing of the muscular compartment, with a reexploration at 24–48 h [16]. We observed open surgery was performed in our study as a salvage therapy in uncontrolled patients after maximal medical treatment failure and is not associated in univariate analysis with a good outcome. In contrast, even when there is no significant statistical association, embolization seems to be useful for the investigation of active bleeding and its treatment. In a recent retrospective cohort of 36 patients presenting with anticoagulation-related soft tissue bleeding, with 21 hematoma located in the ilio-psoas compartment, embolization is efficient and safe for selective arterial embolization. However, the overall mortality in this cohort was 30 %, with a rebleeding rate of 25 % [17]. Thus, even if evidences are scarce, interventional treatment should be considered rapidly in the management of severe IPH.

CT scan is easily and quickly performed and provides useful information such as the localization, evidences for an ongoing bleeding or compression on adjacent structures. Furthermore, another main advantage of this imaging modality is its ability to deduce both origin and arterial or venous source of the bleeding. Therefore, it appears important to perform CT scan with contrast enhancement rapidly in the management of IPH.

One of the main limits of our study is the retrospective character of the cohort and the sample size that did not allowed us to draw solid association through a strong multivariate analysis. The multivariate analysis we herein performed should be taken cautiously given the small population, the importance of hemorrhagic shock in the results and the unavoidable association and overlap of covariates considered in analysis. Because the review of medical charts using keywords is prone to bias, we could have underestimated the true incidence of IPH in the overall population by having omitted the one or other case out. Addition of patients from other ICU so as the screening of surgical ICU could increase the number of patients and strengthen the power of this work. Moreover, we cannot precise the timing for reversal of coagulopathy that may influence the outcome if performed to late.

## Conclusion

We found IPH is an infrequent disease, with a high mortality rate, mostly related to anticoagulation therapy and usually affecting the elderly. Management of anticoagulation-related IPH includes a high rate of no reversal of 38 %. We believe that prospective studies should permit to further identify parameters associated with mortality and to determine whether surgery or embolization is an efficient therapeutic approach.
